# Proteomic analysis of horse hair extracts provides no evidence for the existence of a hypoallergenic Curly Horse breed

**DOI:** 10.1002/clt2.12329

**Published:** 2024-01-29

**Authors:** Bente Janssen‐Weets, Antoine Lesur, Gunnar Dittmar, François Bernardin, Eva Zahradnik, Monika Raulf, François Hentges, Carsten Bindslev‐Jensen, Markus Ollert, Christiane Hilger

**Affiliations:** ^1^ Department of Infection and Immunity Luxembourg Institute of Health (LIH) Esch‐sur‐Alzette Luxembourg; ^2^ Department of Dermatology and Allergy Center Odense Research Center for Anaphylaxis University of Southern Denmark Odense Denmark; ^3^ Quantitative Biology Unit Luxembourg Institute of Health (LIH) Strassen Luxembourg; ^4^ Department of Life Sciences and Medicine University of Luxembourg Esch‐sur‐Alzette Luxembourg; ^5^ Institute for Prevention and Occupational Medicine of the German Social Accident Insurance Institute of the Ruhr‐University Bochum (IPA) Bochum Germany; ^6^ Immunology Allergology Unit Centre Hospitalier Luxembourg Luxembourg

**Keywords:** American Bashkir Curly Horse, Equ c 1, horse allergens, horse proteome, hypoallergenic breeds

## Abstract

**Background:**

The American Bashkir Curly Horse is frequently advertised to horse‐allergic riders and claimed to be a so‐called hypoallergenic breed that elicits fewer symptoms. Previous studies quantifying selected allergens in different breeds did not find a reduced allergen content in Curly Horses. Here, we provide a comprehensive proteomic analysis of horse hair extracts and a molecular analysis of the major allergen Equ c 1 with the aim of identifying differences in the Curly Horse breed that might explain their presumed reduced allergenic potential.

**Methods:**

Horse hair extracts were prepared from Curly and American Quarter Horse breeds, separated by gender and castration status, extracts from other breeds served as controls. Extracts and native Equ c 1 (nEqu c 1) were analyzed by mass spectrometry. IgE‐binding capacities of nEqu c 1 and its recombinant variants were tested by ELISA using sera of patients sensitized to horses. Structures and ligand binding abilities were analyzed by computational modeling and fluorescence quenching assays.

**Results:**

All known respiratory horse allergens are present in hair extracts of Curly and Quarter Horses and share identical allergen‐specific peptides. Lipocalin allergens are the most abundant proteins in horse hair extracts and contain several post‐translational modifications. We identified two new variants of Equ c 1 that have similar IgE‐binding capacities but show structural differences in their binding cavities and altered ligand binding behavior. There are no differences in IgE‐binding of Equ c 1 derived from Curly Horses compared to other horse breeds.

**Conclusion:**

Our data do not support the claim that Curly Horses are less allergenic than other breeds.

## 1 INTRODUCTION

Horses are important contributors to the burden of asthma and allergic rhinitis.^1^ Sensitization to Equ c 1, the major horse allergen, is a highly predictive marker of horse allergy.^2^ Equ c 1 belongs to the protein family of lipocalins and is a relevant allergen in patients with respiratory allergies toward furry animals. Sensitization mainly occurs in individuals polysensitized to similar cross‐reactive allergens shed by other furry species such as cats and dogs.^3^, ^4^ In asthmatic children with a pet allergy, sensitization to Equ c 1 is associated with a more severe disease pattern.^5^ The prevalence of specific IgE (sIgE) to Equ c 1 ranges between 30.6% and 70% among European patients with suspected or diagnosed allergy toward furry animals, whereas a random sample in Sweden revealed a prevalence of 2.1% in the general population.^4^, ^6^, ^7^, ^8^ Horses also cause genuine sensitization; people with regular contact have higher sensitization rates and IgE levels.^1^, ^4^ Horse allergens are widely spread in the environment, and sensitization can occur without direct contact with horses.^1^, ^9^, ^10^ In modern society, horses are popular and primarily used for recreational purposes, competitive sports, and equine‐assisted therapy. The treatment of horse‐allergic patients is limited to drug therapy and the advice to avoid exposure to horse allergens. In the case of horse allergy, evidence of the efficacy and safety of allergen‐specific immunotherapy is insufficient.^11^ Therefore, so‐called hypoallergenic horse breeds, such as American Bashkir Curly Horse (Curly Horse for short), have gained high interest. Breeders and equestrian farms for leisure activities frequently tout the hypoallergenic trait of this breed, mainly associated with its curly, slightly greasy coat. Advertising is often addressed to horse‐allergic patients, claiming that handling Curly Horses is safer than other horse breeds and reduces or does not elicit allergic symptoms.

Studies with horse‐allergic patients and Curly Horses showed that regular contact had no significant effect on lung and nose function in most patients. Initial‐allergic symptoms decreased over time, and clinical tolerance to horses was achieved in some patients.^12^, ^13^ However, controlled clinical trials examining allergic symptoms of characterized patient cohorts upon contact with Curly Horses are missing thus far. Four respiratory allergens are recognized for the species horse (*Equus caballus*) by the World Health Organization/International Union of Immunological Societies (WHO/IUIS) Allergen Nomenclature Sub‐committee. Recent studies have compared the allergen content in horse hair or horse dander and saliva samples of different breeds, including the American Bashkir Curly Horse. The respiratory allergens Equ c 1, Equ c 2, and Equ c 4 were detected in all breeds analyzed. Depending on the study, allergen levels of Curly Horses were either similar or significantly higher compared to other horse breeds.^14^, ^15^, ^16^ Also, air samples collected during grooming showed no differences in allergen content between Curly Horses and the popular horse breed American Quarter Horse.^14^ As these studies relied on antibody‐based detection of horse allergens, variations in allergen sequences might not have been detected. To search for a molecular explanation that could illuminate the presumably lower allergenic potential of Curly Horses, we investigated the composition of horse hair extracts derived from the American Bashkir Curly Horse and the American Quarter Horse by comparative proteomic analysis. Furthermore, we provide an extensive molecular analysis of the major allergen Equ c 1 to elucidate differences among horse breeds and gender.

## 2 MATERIAL AND METHODS

### 2.1 Sampling and protein extract preparation of horse hair

Horse hair was sampled and prepared as part of a previous study.^14^ Briefly, body hair, excluding mane and tail hair, was collected from a currycomb after grooming the horses. Hair was sampled in the early spring of 2016/2017 in 25 stables across Germany, and horse hair protein extracts were prepared. Extracts were stored in aliquots at −80°C, including a Breed/Gender Mix prepared by combining horse hair extracts of 193 individual horses from 32 different breeds.

Breed‐ and gender‐specific extract blends were prepared from American Bashkir Curly Horses and American Quarter Horses living at the same ranch in separate stables under similar conditions. Per breed, horse hair extracts of four males, three castrated males, and 12 females were combined. Extract blends contained 100 μg of protein from each individual.

Horse hair extracts from individual horses of different breeds and gender, from which native Equ c 1 (nEqu c 1) was purified, were freshly prepared, as previously described, with the following modifications.^14^ Horse hair (1 g) was extracted in 40 mL phosphate‐buffered saline at pH 7.4 (PBS) plus 0.05% Tween 20 (PBST). The centrifuged extracts were filtered through a 0.22 μM PVDF membrane (Millipore; Merck).

### 2.2 Production and purification of native and recombinant Equ c 1 variants

Native Equ c 1 was purified from freshly prepared horse hair extracts by affinity chromatography using a HiTrap NHS column (GE Healthcare) coupled with polyclonal antibodies raised against recombinant Equ c 1.0101 as previously described.^14^

Recombinant proteins were produced using synthetic cDNA sequences coding for the mature proteins Equ c 1.0102 (National Center for Biotechnology Information [NCBI] Accession Number [AC]: XP_001489373.1, amino acid residues [AA]: 16–187) and Equ c 1.0201 (AC: XP_001490299.3, AA: 47–218). Codons were optimized for expression in *E.coli* Rosetta‐gami™ 2(DE3) (Novagen^®^ by Merck), a six histidine tag was added at the C‐terminal end, and cDNAs were cloned into vector pET‐21d(+) by GenScript Biotech B.V. Recombinant proteins were purified by immobilized metal ion affinity chromatography (HisTrap HP, GE Healthcare) under native conditions according to the manufacturer's instructions. Recombinant Equ c 1.0101 was further purified by adding an anion exchange chromatography with a Resource Q column (GE Healthcare). The protein was eluted using a linear gradient of 0–500 mM NaCl in 20 mM TRIS‐HCl, pH 7.2. Eluted fractions obtained from both chromatography protocols and containing the native or recombinant proteins were pooled, dialyzed against PBS, and concentrated using Amicon Ultra Centrifugal Filter Units with a 3 kD molecular weight cut‐off (Millipore, Merck).

The purity of the protein solutions was verified by sodium dodecyl sulfate‐polyacrylamide gel electrophoresis (SDS‐PAGE) under reducing conditions as previously described, followed by silver staining (Pierce™ Silver Stain Kit, Thermo Fisher Scientific) according to the manufacturer's protocol.^17^

### 2.3 Analysis of IgE‐binding to horse dander extract and native and recombinant Equ c 1 variants by ELISA

Ten sera of patients sensitized against horse dander were used to test sIgE binding to nEqu c 1 from different breeds. Patients had been recruited at the National Unit of Immunology‐Allergology at the Center Hospitalier de Luxembourg in the frame of previous studies. Ethical approval was obtained from the National Committee for Medical Research Ethics (CNER 2010 01/06 and 2013 07/04), and informed consent was obtained for all subjects. Quantification of sIgE to horse dander (e3), dog dander (e5), and cat dander (e1) was performed with ImmunoCAP (Thermo Fisher Scientific).

Twenty‐four sera of patients with a positive skin prick test (SPT) to a commercial horse dander extract (ALK‐Abelló Nordic A/S) and sIgE to horse dander (mean 15.4 kU/L, range 0.9–69.6 kU/L) were obtained from the serum bank at the Allergy Center of the Odense University Hospital, Denmark. All patients gave written permission for use in scientific protocols in an anonymized way.

Specific IgE‐binding was analyzed by ELISA as previously described.^17^ The proteins were coated at a concentration of 5 μg/mL in PBS. Patient sera were diluted 3–6‐fold in PBS with 3% bovine serum albumin. Nine negative controls with sIgE to mites and/or pollen were tested alongside the rEqu c 1 variants. The average of the negative controls plus two times the standard deviation was used to calculate a cut‐off. Statistical analysis was performed using the Friedman test with Dunn's multiple comparison tests (*p* value > 0.05).

### 2.4 2D‐gel electrophoresis

Native Equ c 1 purified from the Breed/Gender Mix horse hair extract was prepared (2‐D Clean‐Up Kit, GE Healthcare) for two‐dimensional (2D)‐gel electrophoresis that was performed with immobilized pH gradient strips using the PROTEAN i12 isoelectric focusing system (Bio‐Rad Laboratories) as previously described.^17^ Gels were stained with SYPRO Ruby protein gel stain (Invitrogen, Thermo Fisher Scientific) according to the manufacturer's instructions. Images were recorded using the Amersham typhoon gel and blot imaging system (Amersham Typhoon 5, Cytiva).

### 2.5 Liquid chromatography with tandem mass spectrometry (LC‐MS/MS)

Native Equ c 1 samples, separated by 2D‐gel electrophoresis, were prepared according to the in‐gel digestion protocol by Grosch et al.^18^ Briefly, stained protein spots were excised from the gel, cut into small pieces, destained, dehydrated, and dried. Gel pieces were rehydrated in 10 mM dithiothreitol in ammonium bicarbonate 100 mM for 30 min at 55°C and then alkylated for 30 min in the dark with 50 mM iodoacetamide in ammonium bicarbonate 100 mM. Proteolysis was performed by incubating the gel pieces in a trypsin/lysC mixture (Promega). The resulting peptides were extracted and pooled.

Horse hair extract samples were purified using the SP3 on‐bead method, according to Hughes et al.^19^ The samples were clarified by centrifugation, and the cysteine disulfide bonds were reduced with 10 mM dithiothreitol in ammonium bicarbonate 50 mM for 30 min at room temperature (RT). Samples were then alkylated with 30 mM iodoacetamide in ammonium bicarbonate 50 mM for 30 min in the dark at RT. SP3 beads (Thermo Fisher Scientific) were added to the samples. Formic acid and neat acetonitrile (50% of the final volume) were added to capture the protein onto the beads. Beads were washed twice with a 70% ethanol‐water solution, then with acetonitrile, and finally dried. The beads were suspended in 20 μL of a proteolysis solution containing a trypsin/LysC mixture (Promega) in ammonium bicarbonate 50 mM and incubated overnight at 37°C. After digestion, the peptides were captured on the beads by adding pure acetonitrile. The beads were washed three times with acetonitrile, and peptides were eluted from the beads with a solution of 2% dimethyl sulfoxide in water. Finally, all samples were vacuum‐dried and suspended in an aqueous buffer containing 1% acetonitrile and 0.05% trifluoroacetic acid.

The liquid chromatography with tandem mass spectrometry (LC‐MS/MS) setup consisted of a Dionex Ultimate 3000 RSLC chromatography system configured in a column switching mode coupled with a *Q* Exactive‐Plus mass spectrometer (Thermo Fisher Scientific). Liquid Chromatography and mass spectometry (MS) acquisition were performed as previously described.^18^ Samples were eluted at a flow rate of 0.3 μL/min for 33 min.

The recorded mass spectrometric data were analyzed using the MaxQuant v 2.1.3.0 software package with the NCBI *Equus caballus* protein database (October 07, 2022, 78223 entries) for relative peptide quantification and the horse proteome reference database (UP000002281, downloaded 18.10.2022) for post‐translational modifications (PTMs). Variable modifications were set to N‐terminal acetylation and methionine oxidation, while the fixed modifications were set to carbamidomethylation of cysteines. Matches between runs were allowed. The quantitative data were normalized by the label‐free quantification algorithm. Identifications were filtered with a false discovery rate of 0.01. Protein groups were filtered with a minimum of 2 razor + unique peptides detected. Relative peptide quantification of allergens and their variants in horse hair extracts was performed by analyzing only unique peptides that were specific to the corresponding variant and found in every sample. For PTMs, the analysis of secondary peptides and the detection of dependent peptides was allowed. The resulting files were analyzed using *R.* ^20^

### 2.6 Fluorescence quenching assays

Fluorescence quenching assays were performed as previously described, including the ligand test panel.^21^ Test solutions contained equimolar concentrations (5 μM) of an Equ c 1 variant and the fluorescent probe n‐phenyl‐1‐naphthylamine (1‐NPN) in a final buffer composition of 5% methanol in PBS. All measurements were performed at an excitation wavelength of 337 nm and an emission at 396 nm on a SpectraMax iD3 (Molecular Devices) fluorescence reader.

Ligand binding to 5 μM Equ c 1.0102 was analyzed through the competitive displacement of the fluorescent probe 1‐NPN by adding 50 μM ligand. The applied 1‐NPN concentration equals to the EC_50_ value, at which half of the allergen binding sites are saturated with 1‐NPN (EC_50_ = 5 μM; 95% CI = 4.1–5.7 μM).

The strongest binding ligands, 3,7‐dimethyl‐1‐octanol and 1‐decanol, were analyzed by dose‐dependent titration curves. Twelve 2‐fold dilutions of 0–50 μM ligands were titrated to 1‐NPN‐Equ c 1.0102 complexes. Titration curves were analyzed, and ligand dissociation constants (*K*_*i*_) were calculated by non‐linear regression using a competitive‐binding model assuming one‐site fit and specific binding (least squares fit, no weighting, outlier elimination with *Q* = 1%).

### 2.7 Analytical software

Statistical analysis and graph design were performed with GraphPad Prism Version 9.3.1 for Windows (GraphPad Software, www.graphpad.com). Clustal Omega via EMBL‐EBI Web Services was used for multiple sequence alignments.^22^, ^23^ In silico structure modeling of proteins was performed using AlphaFold2.^24^ Protein structures were visualized using The PyMOL Molecular Graphics System, Version 1.6.x Schrödinger. Protein surface areas and volumes were calculated using CASTp (Computed Atlas of Surface Topography of proteins) Analysis.^25^

## 3 RESULTS

### 3.1 No difference in IgE‐binding of major allergen Equ c 1 derived from Curly Horses compared to other horse breeds

**FIGURE 1:**
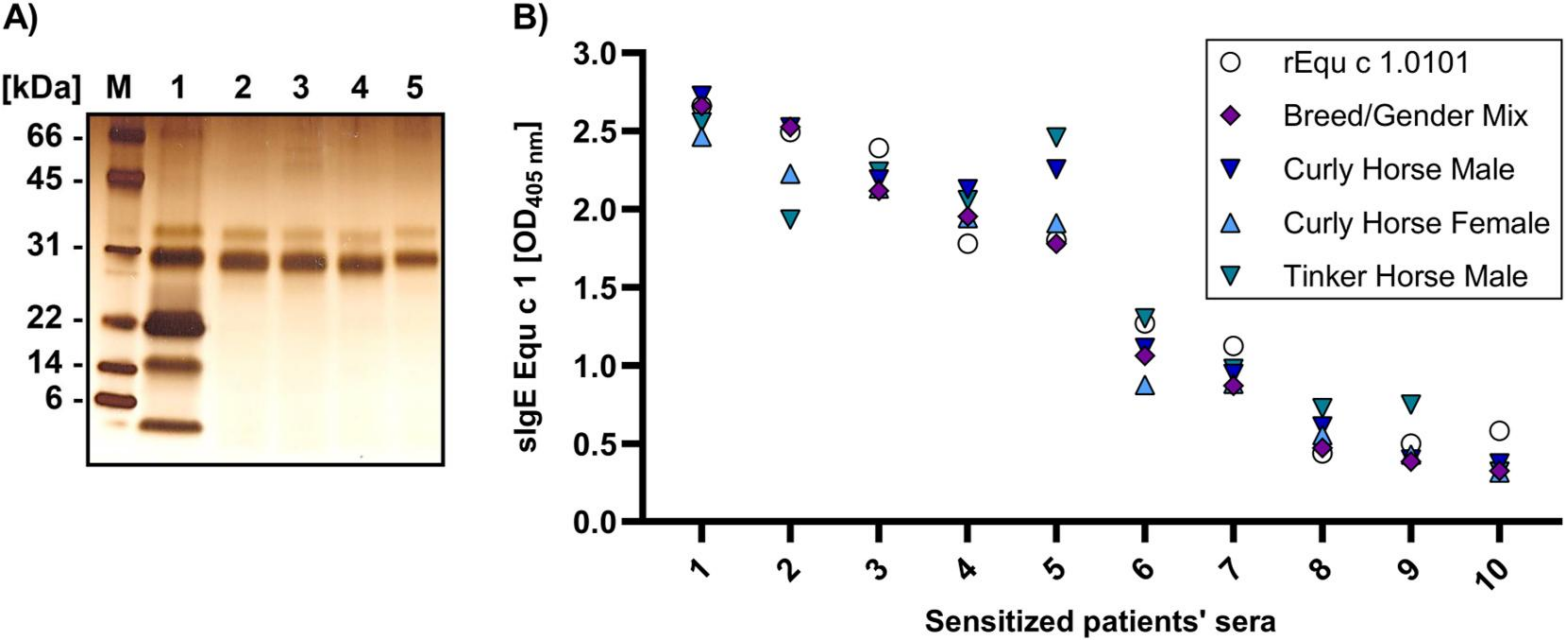
No difference in IgE‐binding of native Equ c 1 purified from different horse hair extracts. (A) Silver‐stained SDS‐PAGE gel of a horse hair extract Breed/Gender Mix (1) and native Equ c 1 purified from horse hair extracts of a Breed/Gender Mix (2), a Tinker Horse male (3), a Curly Horse male (4) and a Curly Horse female (5). (B) Comparison of IgE‐binding in sera of patients sensitized against horse dander (*x*‐axis, patient sera numbered 1–10; results are ranked by decreasing signal intensity) to native Equ c 1 purified from horse hair extracts of different breeds and genders and recombinant Equ c 1 by ELISA.

Native Equ c 1 was purified from horse hair extracts of different breeds by affinity chromatography using polyclonal antibodies raised against recombinant Equ c 1.0101. The source material was collected as part of a previous study.^14^ Extracts were prepared from a Curly Horse male (stallion), a Curly Horse female (mare), and a Tinker Horse male (stallion). Additionally, a Breed/Gender Mix was created by combining horse hair extracts of 193 individual horses of different genders from 32 breeds. Purified nEqu c 1 presents a molecular weight between 28 and 33 kDa in a silver‐stained SDS‐PAGE gel (Figure 1A).

IgE‐binding to nEqu c 1 from different breeds was analyzed by ELISA. We tested 10 sera of sensitized patients with sIgE levels against horse dander ranging from 12 kU/L to >100 kU/L (Table S1). No significant differences in IgE‐binding to nEqu c 1 from different breeds and genders and recombinant Equ c 1.0101 were found (*p* value > 0.05) (Figure 1B).

### 3.2 Identification of two new IgE‐binding variants of the major horse allergen Equ c 1.0101

**FIGURE 2:**
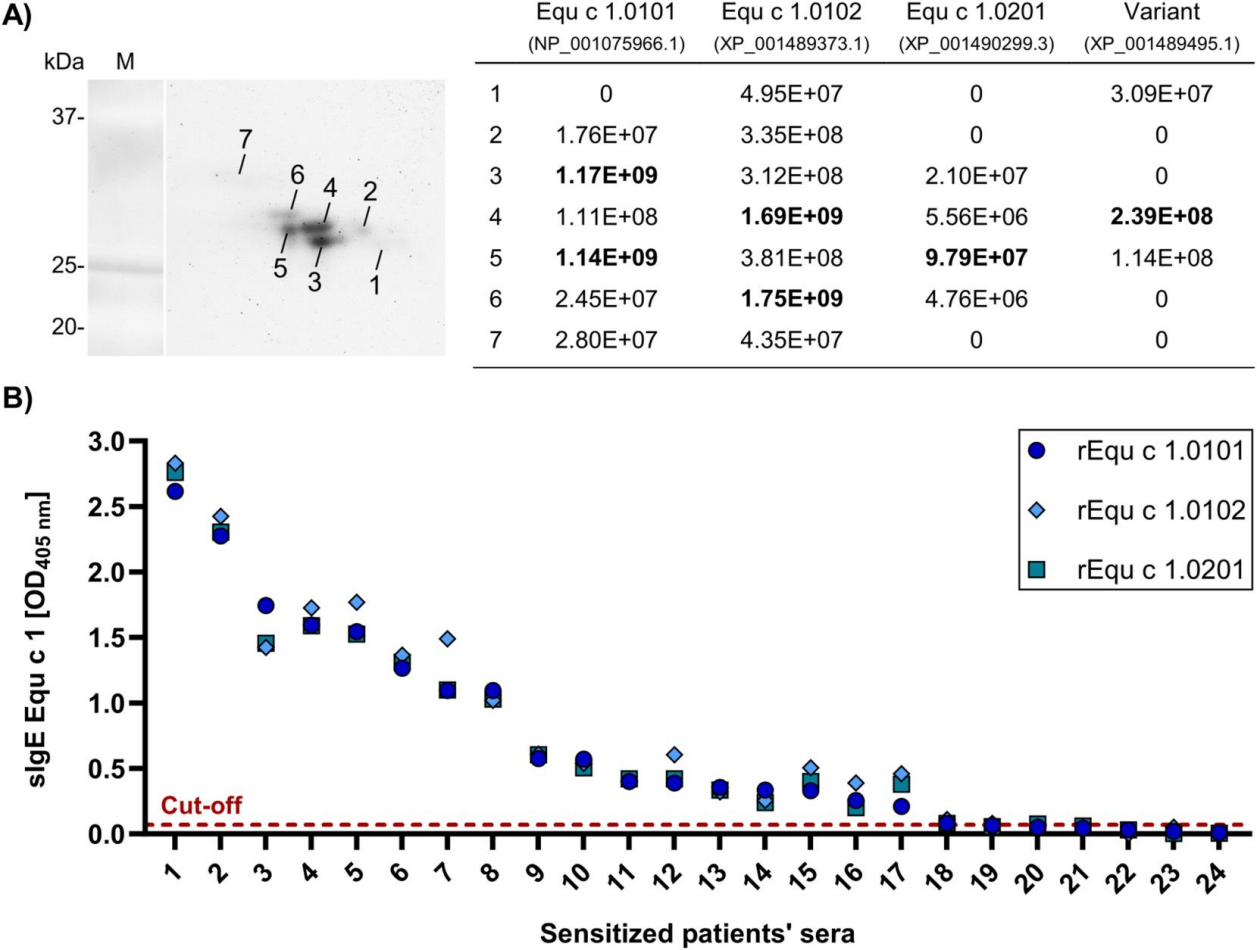
Identification of allergenic variants of the major horse allergen Equ c 1 (A) Localization of major horse allergen Equ c 1 variants by MS in a 2D‐gel of native Equ c 1 purified from a mix of horse hair extracts of different breeds and genders. Stained protein spots were picked and analyzed by LC‐MS/MS. The sum of intensities of unique peptides for Equ c 1 variants was calculated in each spot for relative quantification. Bold numbers represent the highest intensities found for each variant. (B) IgE‐binding capacity of recombinant Equ c 1.0101, Equ c 1.0102, and Equ c 1.0201 analyzed by ELISA using 24 sera of patients sensitized against horse hair/dander extracts. The cut‐off was calculated using the average of 9 negative controls plus two times the standard deviation. Results are ranked and displayed by decreasing signal intensity.

We further investigated the composition of the nEqu c 1 batch purified from the Breed/Gender Mix horse hair extract by proteomic analysis. Native Equ c 1 was separated by 2D‐gel electrophoresis (Figure 2A). Stained protein spots were excised from the gel and analyzed by LC‐MS/MS.

Besides Equ c 1.0101, the only variant officially recognized as an allergen thus far, we found specific peptides of three more variants. Their amino acid sequences have been previously predicted by automated computational analysis derived from genomic sequences and are available in the NCBI database. The two most abundant variants were designated Equ c 1.0102 and Equ c 1.0201 and accepted by the WHO/IUIS Allergen Nomenclature Sub‐Committee as new Equ c 1 variants.

The sum of intensities of unique peptides for the Equ c 1 variants allows their relative quantification and localization in the gel (Figure 2A). All found Equ c 1 variants run at a molecular weight between 27 and 33 kDa. Equ c 1.0101 and Equ c 1.0102 are the two most abundant variants, and unique peptides are present in almost every spot and show the highest intensities among all variants. Equ c 1.0101 predominantly localizes in spots 3 and 5 between 27 and 29 kDa. Equ c 1.0102 shows its highest intensities in spots 4 and 6 between 28 and 30 kDa. Both variants are found in lesser intensities in spot 7 at 31–33 kDa. Equ c 1.0201 is primarily found in spot 5 at 28 kD and generally in lower intensities than other variants. Only one unique peptide of another variant (GenPept reference sequence: XP_001489495.1), showing only 67% amino acid sequence identity to Equ c 1.0101, was found around 28 kDa.

We confirmed the amino acid sequences of the new variants Equ c 1.0102 and Equ c 1.0201 by LC‐MS/MS of the Breed/Gender Mix extract in solution. For Equ c 1.0102, 93.6% sequence coverage was reached (Figure S1A). In total, 30 peptides were found, of which 20 were unique to Equ c 1.0102. For Equ c 1.0201, 84.3% of the sequence was covered by 22 peptides, and 14 were unique (Figure S1B).

We further characterized the IgE‐binding of recombinant Equ c 1.0102 and Equ c 1.0201 by ELISA and compared it with the well‐known allergen Equ c 1.0101. An additional 24 sera from sensitized patients with a positive SPT to horse dander (3–20 mm) and sIgE against horse dander (0.9–69.6 kU/L) were analyzed (Table S2). The IgE‐binding capacity of all three recombinant Equ c 1 variants was similar for each serum tested (*p* value > 0.05) (Figure 2B).

### 3.3 Equ c 1 variants show structural differences in their binding cavities and altered ligand binding behavior

**FIGURE 3:**
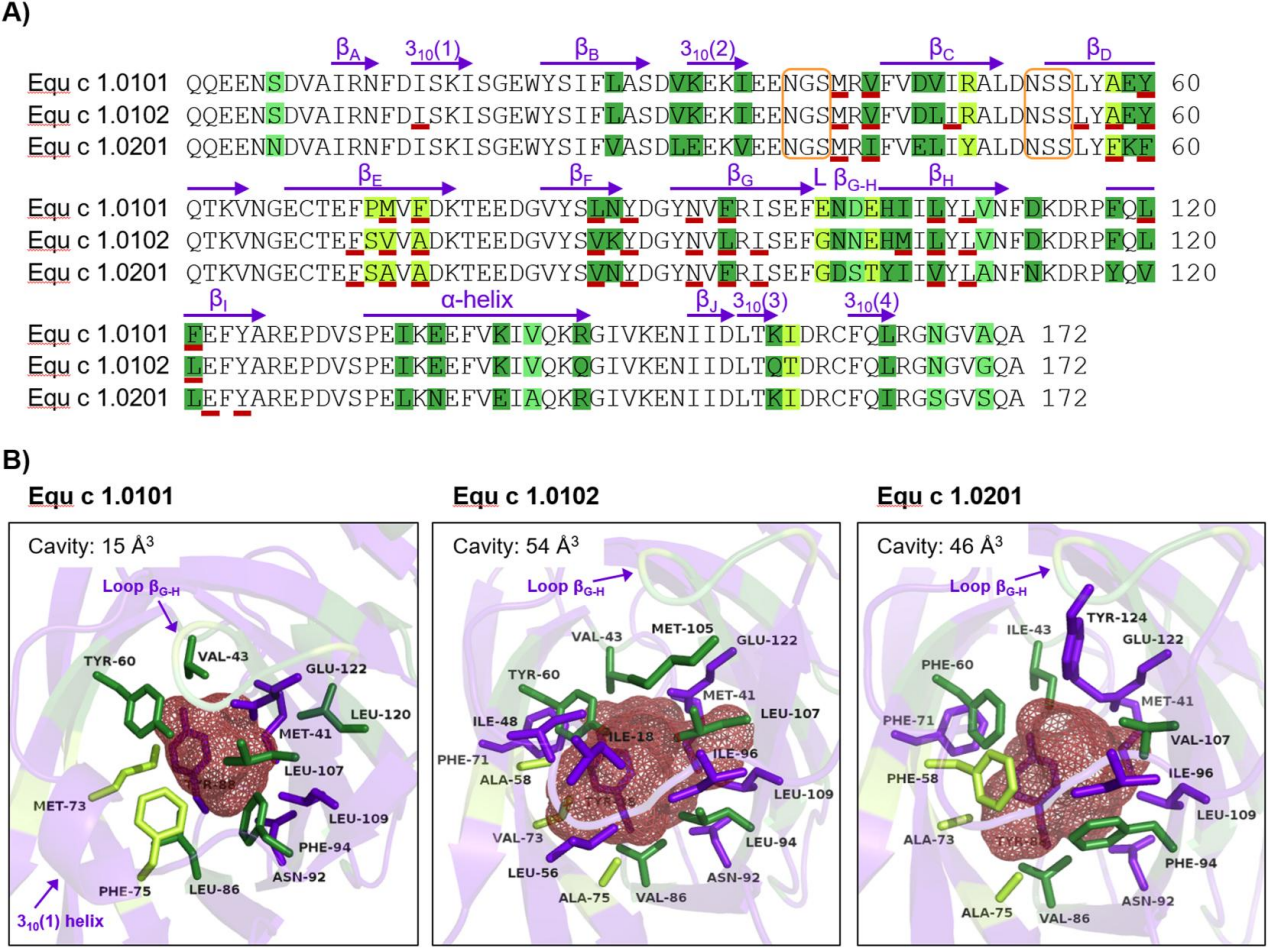
Structural differences in major horse allergen Equ c 1 variants. (A) Multiple sequence alignment of Equ c 1 variants without signal‐ or pro‐peptides. Secondary structures of Equ c 1.0101 (*β*‐sheets, *α*‐ and 3_10_‐helices) are indicated by purple arrows above the sequence. Substitutions in amino acid positions between the variants are highlighted in green, whereas dark green represents strongly similar residues and bright green variable residues. AA forming the protein cavities are underlined in red. N‐linked glycosylation motifs are framed in orange. (B) Cartoon representation of the binding cavities of Equ c 1.0101, Equ c 1.0102, and Equ c 1.0201. A red mesh illustrates the protein cavity spaces, and their calculated volumes are stated in the upper left corners, respectively. AA lining the cavities are represented as sticks. Purple residues are conserved between the variants; dark greens are strongly similar, and bright greens are variable. AA, amino acid residues.

Equ c 1 variants identified in horse hair extracts were further characterized regarding their molecular structure and ligand binding. The isoforms Equ c 1.0101 and Equ c 1.0102 show 91.3% identity in their mature protein amino acid sequences, whereas Equ c 1.0201, with 80.2% amino acid sequence identity to Equ c 1.0101, is classified as an isoallergen (Figure 3A). Variants of lipocalin allergens also exist in other furry animals such as bovine (Bos d 2), guinea pig (Cav p 1), rat (Rat n 1), and mouse (Mus m 1).^26^ Lipocalin allergens can bind small signaling molecules, such as odorants or pheromones, in their hydrophobic binding pocket.^21^, ^27^, ^28^ The conservation of lipocalin variants in several species throughout evolution might relate to their function as transport proteins. Substitution of only a few AA that shape the ligand binding pocket can result in altered ligand binding affinity and specificity.^29^, ^30^ Therefore, we analyzed the tertiary structure of the Equ c 1 variants by computational modeling. Indeed, we observed the most amino acid substitutions among the Equ c 1 variants around the cavity lining area (Figure 3B). The cavity volume of Equ c 1.0101 is over three times smaller than the other variants. The helical structure 3_10_(1) narrows its cavity entrance. It is replaced by a flexible loop in Equ c 1.0102 and Equ c 1.0201. Although this tetrapeptide is identical in all three variants, it organizes into different shapes, probably defined by its neighboring structures. The loop at position 100–103 connecting *β*‐sheet G and H is part of the largest peptide of contiguous variability of AA between the three sequences. It partly covers the cavity entrance of Equ c 1.0101. The AA methionine 73 and phenylalanine 75 also restrict the cavity space in Equ c 1.0101. Much smaller residues in the other variants, such as valine and alanine, substitute them. Phenylalanine residues at positions 58 and 94 are capping the cavity space in Equ c 1.0201. In contrast, these positions are occupied with alanine and leucine in Equ c 1.0102 and facilitate an open cavity. The residue positions 43, 60, 94, and 107 seem well conserved. Their amino acids are present at the surface area of the cavity in all three Equ c 1 variants and also line the hydrophobic binding pocket of the major mouse allergen Mus m 1, where they are in contact with the pheromone ligand upon binding.^27^, ^31^

**FIGURE 4:**
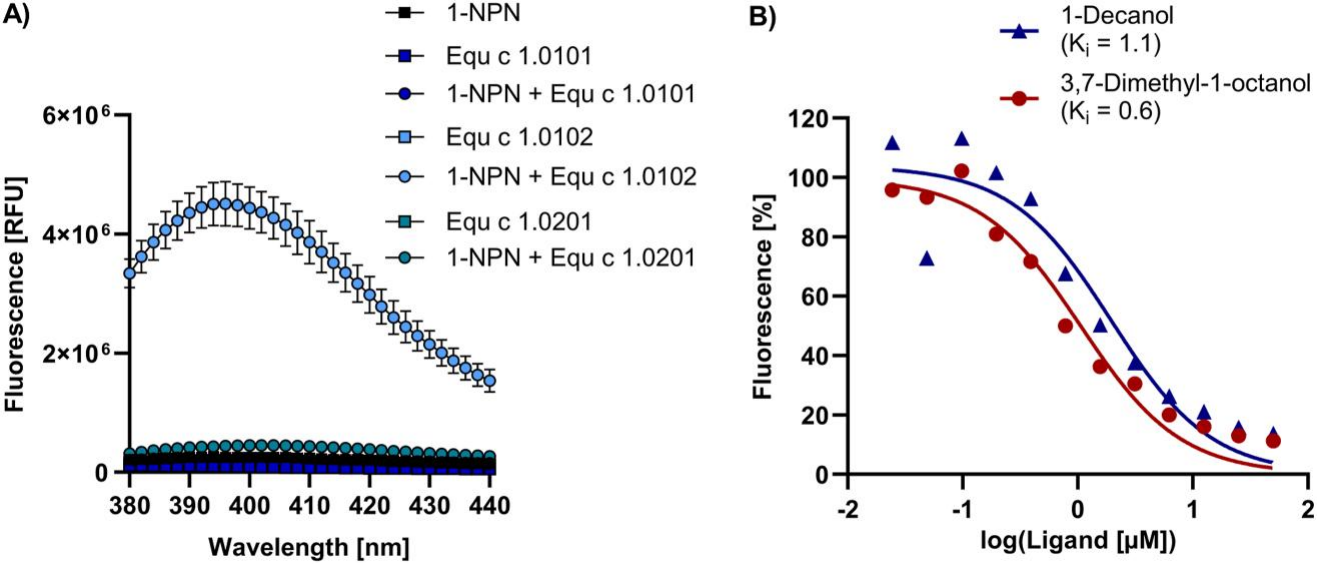
Ligand binding analysis of Equ c 1 variants using a fluorescence quenching assay with 1‐NPN. (A) Emission spectra of Equ c 1 variants combined with the fluorescent probe 1‐NPN at an equimolar concentration of 5 μM and an excitation wavelength of 337 nm. (B) Fluorescence‐inhibition of 1‐NPN bound to Equ c 1.0102 by dose‐dependent titration of 0–50 μM 1‐decanol or 3,7‐dimethyl‐1‐octanol. Ligand dissociation constants (*K*_*i*_) were calculated by non‐linear regression using a one‐site fit specific binding model. 1‐NPN, n‐phenyl‐1‐naphthylamine.

We analyzed the binding of the fluorescent probe 1‐N‐phenyl‐1‐naphthylamine (1‐NPN) to the three Equ c 1 variants. 1‐NPN emits fluorescence when bound to the polar residues of the lipocalin binding pocket.^28^ Only variant Equ c 1.0102 bound 1‐NPN with a dissociation constant of 6.4 μM (95% CI = 5.8–7 μM), whereas no fluorescence signal was detected for variants Equ c 1.0101 and Equ c 1.0201 (Figure 4A). Alternate testing of the fluorescent probes 1‐aminoanthracene (1‐AMA) or 8‐anilino‐1‐naphthalensulfonic acid (1,8‐ANS) and 1‐NPN at pH 9 instead of pH 7.5 did not enhance binding in any of the three variants (data not shown). Out of 38 tested ligands comprising odorants, pheromones, and steroids, the fatty alcohols 3,7‐dimethyl‐1‐octanol and 1‐decanol were most effective in displacing 1‐NPN from the binding pocket of Equ c 1.0201 with dissociation constants of 0.6 and 1.1 μM, respectively (Figure 4B). The odorant 3,7‐dimethyl‐1‐octanol has been previously identified as a strong binding ligand of nEqu c 1, where Equ c 1 was investigated as a potential semiochemical carrier in the horse.^28^ The three Equ c 1 variants showed marked structural differences in the ligand binding cavities, translating into different binding properties.

### 3.4 Known respiratory horse allergens are present in hair extracts of the Curly and the Quarter Horse breed and share identical, allergen‐specific peptides

**FIGURE 5:**
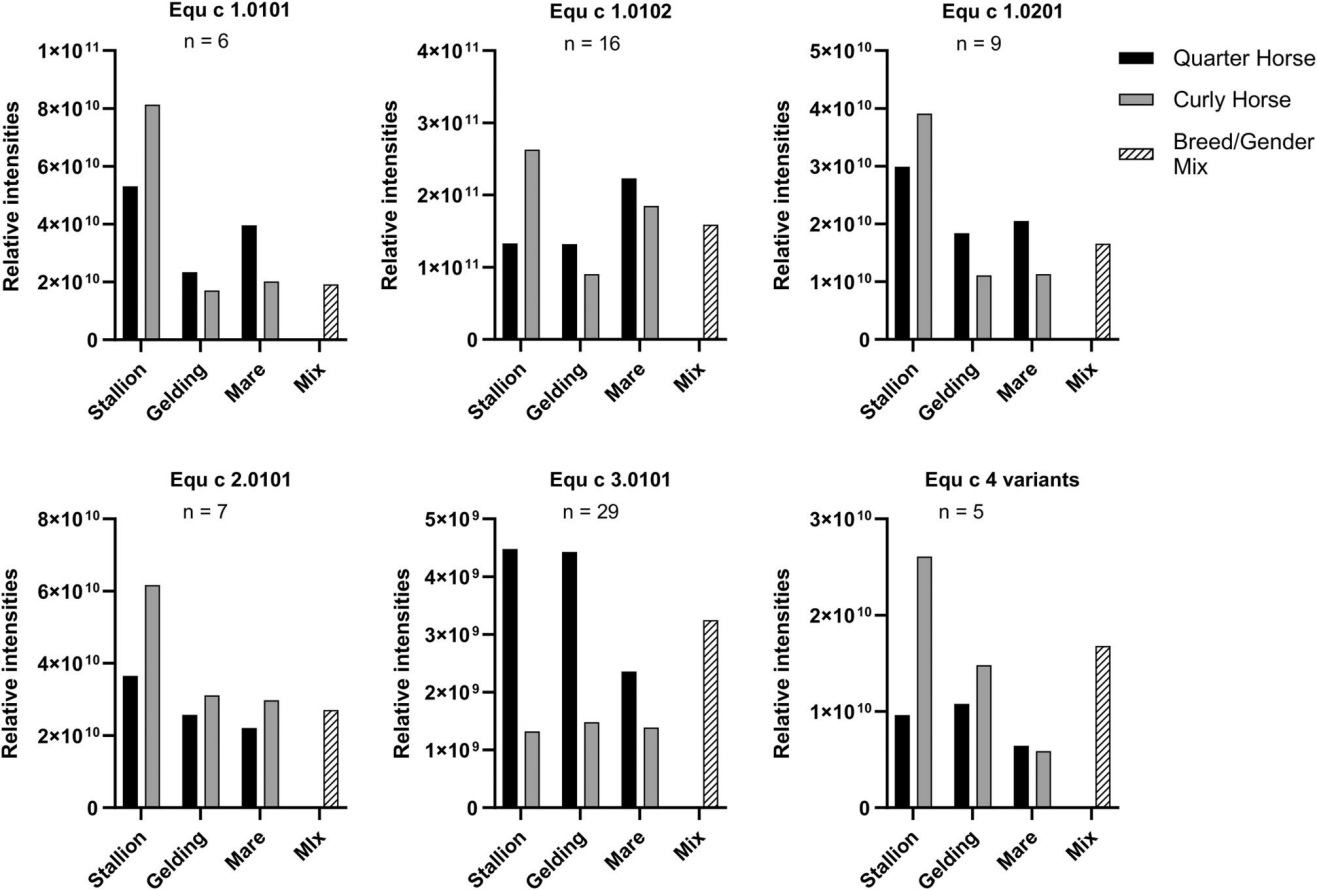
Relative peptide quantification of allergens and their variants in horse hair extracts of the Quarter Horse breed (black bar), Curly Horse breed (gray bar), and a Breed/Gender Mix (patterned bar). For each breed, horse hair extracts of four males (stallion), three castrated males (gelding), and 12 females (mare) were combined and analyzed by mass spectrometry. The Breed/Gender Mix is a combination of horse hair extracts from 193 horses of different breeds and genders. The sum of relative peptide intensities is graphed for each protein. Only unique peptides that are specific to an allergen variant and were found in every sample were included in this analysis. Specific peptides for Equ c 4 variants were combined since they share 98.7% amino acid sequence identity. *n* = number of unique peptides.

To further investigate differences between the Curly Horse and other breeds, we analyzed horse hair protein extracts using MS. We compared the proteome of the Curly Horse and the Quarter Horse breed, focusing on respiratory allergens and their variants. We differentiated between gender and castration status. Per breed, we combined horse hair extracts of four males (stallions), three castrated males (geldings), and 12 females (mares) for MS analysis. The previously described Breed/Gender Mix containing several different hair extracts was used as a control. We found specific peptides of all known respiratory allergens in every sample. The relative intensities of unique allergen‐specific peptides were summed and compared between samples to analyze the relative allergen quantity in different breeds and genders (Figure 5). The sum of relative peptide intensities derived from horse allergen Equ c 1 variants, Equ c 2.0101, and Equ c 4 variants in extracts of Curly and Quarter Horse geldings and mares show some variability compared to each other as well as to the Breed/Gender Mix. Stallion extracts tend to contain higher allergen content than mares, except for Equ c 1.0102 in Quarter Horse extracts and Equ c 3.0101 in Curly Horse extracts. Among stallion extracts, the Curly Horse breed shows an increased allergen content compared to the Quarter Horse breed in five out of six allergens. The most notable difference between breeds is found for allergen Equ c 3.0101, a serum albumin protein abundant in blood plasma. All Curly Horse hair extracts have a relatively low Equ c 3.0101 content compared to Quarter Horses and the Breed/Gender Mix.

Similarly, upon analyzing other proteins abundant in blood plasma, these were either not detected or showed decreased quantities in hair extracts of Curly Horses compared to Quarter Horses (Table S3). These include immunoglobulin (Ig) G and IgM, antithrombin‐III, serotransferrin precursor, and apolipoprotein A‐I. Equally decreased were the calcium‐activated chloride channel expressed in epithelial and endothelial cells, cathepsins (cysteine and aspartyl proteases), and synaptophysin‐like protein 1. Conversely, proteins with increased quantities in hair extracts of Curly Horses compared to Quarter Horses are predominantly found in the skin, such as trichohyalin, a protein highly expressed in cells of hair follicles, desmocollin‐3, a cadherin in epithelial cells required for cell‐adhesion, as well as cystatin‐A and keratin, structural proteins of keratinocytes. Highly abundant in Curly Horse hair extracts were also BPI fold‐containing family A member 2‐like, a lipopolysaccharide‐binding protein of the innate immune response against bacteria, zinc‐alpha‐2‐glycoprotein is found in serum and sweat and stimulates lipolysis as well as the secretoglobin mammaglobin.

The comprehensive analysis of the horse hair proteome revealed that respiratory horse allergens constitute the most abundant proteins in horse hair extracts of both breeds and the Breed/Gender Mix; ahead are lipocalin Equ c 1 variants and lipocalin Equ c 2.0101, followed by the latherin Equ c 4 (Table S3). Proteins belonging to the secretoglobin superfamily are also highly abundant. Secretoglobins are recognized as major allergens in the cat and rabbit but not yet in the horse.

### 3.5 Lipocalin horse allergens contain peptides with several post‐translational modifications

As we did not find any differences in the allergen sequences among breeds, we investigated PTMs as a cause for the presumed hypoallergenicity of the Curly Horse. We further used the data obtained by MS to analyze modifications of peptides found in horse hair extracts. In total, 27 different modifications were found (Table S4). Fourteen of those modifications were neglected because the sample treatment could have caused their presence. The protein family of lipocalins, among them the major horse allergens, contained numerous peptides with various modifications. Di‐Methylation was by far the most intense modification found on a lipocalin peptide present in all analyzed extracts. The di‐methylated peptide was 12 to 35‐fold more abundant than its unmodified form. The corresponding protein (A0A3Q2HUS7) shows 77% amino acid sequence identity to Equ c 1.0101.

The most common modification among lipocalin allergens and the latherin allergen Equ c 4 was the formation of acetaldehyde adducts, predominantly found in hair extracts of Curly Horse stallions and geldings. Acetaldehyde modifications were of low intensity, and the modified form occurred on average in less than 1% of peptides. One exception is a peptide containing a glycosylation site of Equ c 1.0101 that was two times more abundant in its modified than unmodified form. The relevance of these modifications on the allergenicity of proteins needs to be clarified.

Formylation of peptides was another relatively abundant modification of generally low intensity among lipocalins in most extracts. Two similar peptides located near the C‐terminal end of Equ c 1.0101 and Equ c 1.0102 showed relatively high ratios of formylated to unformylated peptides ranging from 6% to 70%, depending on the extract.

Several other modifications on peptides of respiratory horse hair allergens appeared sporadically among extracts with generally low intensity, such as acetylation, methylation, tri‐methylation, phosphorylation, tryptophan degradation (Kynurenine pathway), disulfide bonds, sulfide‐, didehydro‐, dethiomethyl‐ and propionyl‐modifications.

## 4 DISCUSSION

The existence of so‐called hypoallergenic animals and their suitability as pets or companion animals are often a matter of great interest and discussion.^14^, ^32^, ^33^, ^34^, ^35^ The present study aimed to investigate differences in protein and allergen content of hair extracts from the Curly Horse, claimed as hypoallergenic, and the Quarter Horse. Whereas previous studies have focused on allergen quantification, we pursued a proteomics strategy to identify new proteins and/or differences in allergen amino acid sequences that might have been missed using polyclonal antibody detection. Hair extracts of the presumably hypoallergenic horse breed American Bashkir Curly Horse contained the same respiratory allergens as other horse breeds. Allergen‐specific peptides were found in all Curly Horse hair extracts analyzed, irrespective of gender and castration status. IgE‐binding to the major horse allergen Equ c 1 derived from Curly Horses did not differ from Equ c 1 derived from other horse breeds. We confirmed the existence of two new IgE‐binding Equ c 1 variants in all horse hair extracts. These variants showed structural differences in their binding cavities and altered ligand binding behavior. Proteomic analysis revealed that respiratory allergens were the most abundant proteins in horse hair extracts. Ahead was the protein family of lipocalins containing peptides with several PTMs.

Our data are in line with previous studies on so‐called hypoallergenic animals. Until now, there is no scientific evidence for the existence of hypoallergenic cat, dog, and horse breeds.^14^, ^35^ So far, studies addressing the topic of hypoallergenic breeds have used antibody‐based techniques to quantify allergens in the dust of homes or the animals' coats, saliva, and urine. “Hypoallergenic” dog and horse breeds were not associated with significantly reduced allergen content.^14^, ^15^, ^16^, ^36^, ^37^

The material analyzed in the present study was obtained in a larger project where horse dander antigen, Equ c 1, and Equ c 4 levels were previously quantified by ELISA in horse hair extracts of 32 breeds.^14^ Allergen concentrations varied strongly in individual horses, but median Equ c 1 levels were 2.6‐fold, and Equ c 4 levels were 3.1‐fold higher in Curly Horses compared with Quarter Horses. Overall, stallions had significantly higher allergen content compared with mares in the ELISA analyses. This high allergen content in Curly Horses was puzzling. Therefore, in this follow‐up study, we decided to focus on a proteomic analysis to identify potential differences in horse allergen primary structures and the overall protein composition in horse hair extracts of different breeds.

Our current study used pooled individual horse hair extracts divided by breed, gender, and castration status, representing only part of the original cohort. The present cohort included Curly and Quarter Horses living at the same ranch in separate stables under similar conditions. When neglecting gender and castration status, relative peptide quantification showed that Quarter and Curly Horse hair extracts contained similar quantities of Equ c 1 variants. Curly Horse extracts had higher Equ c 4 levels than Quarter Horse extracts. Overall, Curly Horse stallion extract showed high allergen content compared to all extracts analyzed, confirming our previous results obtained by antibody quantification.

Two other recent studies quantified Equ c 1, Equ c 2, and Equ c 4 levels in horse dander and saliva samples among 10 horse breeds, including Curly and Quarter Horses.^15^, ^16^ Their results showed high intra‐ and inter‐breed variations as well. No significant differences in allergen contents in horse dander samples between breeds were found. Equ c 4 levels in horse dander and saliva samples and Equ c 2 levels in saliva samples were significantly higher in stallions.

The serum albumin allergen Equ c 3 has not been subjected to environmental allergen quantification studies in dust or horse material thus far. Here we observed reduced Equ c 3 content in horse hair extracts of Curly Horses compared to Quarter Horses. Similarly, we found reduced levels of other blood plasma proteins in Curly Horse hair extracts compared with Quarter Horse extracts. In contrast, Curly Horse hair extracts contained higher contents of skin proteins, such as trichohyalin. Trichohyalin is a protein highly expressed in the cells of hair follicles. It is expressed in several dermatological disorders and is associated with uncombable hair syndrome.^38^, ^39^ The coat of Curly Horses manifests with various curl phenotypes ranging from loose waves up to dense micro curls, sometimes accompanied by hypotrichosis.^40^, ^41^ In contrast, Quarter Horses presented with straight, short hair. Since we obtained the material for the hair extracts by grooming, hair structure could have influenced the proteomic results, for example, a dense curly hair coat could have impeded direct skin contact with the currycomb, thus explaining lower intensities of plasma proteins in Curly Horse hair extracts.

Our previous study revealed that the Curly Horse's hair pattern did not influence the release of Equ c 1, Equ c 4, and overall antigen content into the air during grooming compared with Quarter Horses.^14^ Data on Equ c 3 are not available. If there were indeed reduced Equ c 3 content in Curly Horses, it would most likely not be relevant for most horse‐allergic riders since 76%–100% are sensitized against the major allergen Equ c 1, and Equ c 3 is a minor allergen.^26^

To our knowledge, this is the first comprehensive proteomic analysis of proteins found in horse hair extracts. Respiratory allergens constituted the most abundant proteins in horse hair extracts, such as the lipocalin Equ c 1 variants, lipocalin Equ c 2, and latherin Equ c 4, followed by the protein family of secretoglobins. The most abundant horse secretoglobins share 12%–57% amino acid sequence identity with chains of the major cat allergen Fel d 1 and rabbit allergen Ory c 3. Nothing is known so far about their allergenic potential.

We identified two new IgE‐binding variants of Equ c 1. The three variants show structural differences in their ligand binding cavities altering their ability to interact with the fluorescent probe 1‐NPN. We confirmed 3,7‐dimethyl‐1‐octanol as a strong binding ligand for recombinant Equ c 1.0102. This odorant has been previously identified as a strong binding ligand of nEqu c 1.^28^ Due to its ligand binding ability and the high amino acid sequence identity to pig salivary lipocalin (60%) and rodent major urinary proteins (47% to Mus m 1), proven semiochemicals, Equ c 1 is proposed to play a role in chemical communication as well.^21^, ^28^ A significant number of lipocalin variants exist in mouse urine. Individuals use these major urinary proteins to create a distinctive identity signal.^42^ The variants show specific differences in the binding and release of volatile ligands.^42^, ^43^ The binding of small hydrophobic ligands has been demonstrated for several lipocalin allergens from other furry animals, but their species‐specific function remains elusive.^21^ Unfortunately, in our study, the retrospective use of patient sera did not allow to collect fresh blood samples for in vitro cellular assays such as the basophil activation test to analyze a potential impact on allergenicity by different ligands and Equ c 1 variants.

It is striking that lipocalins in horse hair extracts contained many PTMs for their relatively small molecular weight of 16–25 kDa. PTMs comprise the cleavage of peptide bonds and the covalent addition or modification of functional groups on the protein's amino acid side chains or C‐ and N‐termini. PTMs influence protein function and interaction with other molecules by modifying the protein's biophysical and chemical properties. It is well known that Equ c 1 and its variants are glycosylated.^44^, ^45^ Carbohydrate moieties, attached to allergens or by themselves, can be targets for IgE‐antibodies.^46^ Not much is known about the impact of other PTMs on the allergenic potential of proteins. We found PTMs that can influence the structure. Sulfides, essential for the formation of disulfide bonds, have a significant impact on the secondary and tertiary protein structure. Methylation, phosphorylation, and acetylation can regulate protein stability.^47^ Stability is a common property of allergens.^48^ We observed several modifications on peptides containing amino acids that line the binding cavities. It is possible that PTMs could influence the binding and release of ligands.

We found acetaldehyde adducts on several peptides of lipocalin allergens and Equ c 4. Acetaldehyde can form stable adducts with proteins that alter their structure and impair protein function.^49^ Acetaldehyde is a volatile organic compound and a priority pollutant due to its ubiquity and carcinogenic potential.^50^ It irritates the skin, eyes, and respiratory tract.^51^ In the indoor environment, acetaldehyde sources include building and wood‐based materials, wall paints, candles, ethanol, tobacco smoke, and leather.^50^

The environment potentially influences protein modifications. Regarding horses and their allergens, the exposome might comprise the stables' microbiome, building material, riding equipment, insecticides, care products, and humans. More research is needed on allergen PTMs and their impact on the human immune system, also considering environmental triggers.

Here, we give an overview of PTMs found in combined horse hair extracts of individual horses matched by breed and gender. To make a statistical statement on the frequency and relevance of specific modifications in different horse breeds and genders, analyses on individual horse hair extracts and larger sample sizes are needed.

## 5 CONCLUSION

Our data suggest that handling Curly Horses is not safer for allergic patients compared to other breeds. Patients with sIgE to horse dander and respiratory symptoms in the presence of horses are advised to avoid exposure to horses, regardless of the breed. However, a controlled clinical trial with a characterized patient cohort and a control horse breed is needed to make a final statement on the possible benefit of Curly Horses for horse‐allergic riders.

The existence of IgE‐binding lipocalin variants and the multitude of PTMs detected add another layer of complexity to the research on allergenic proteins.

## AUTHOR CONTRIBUTIONS

**Bente Janssen‐Weets**: Conceptualization (equal); formal analysis (equal); investigation (lead); visualization (lead); writing – original draft (lead); writing – review & editing (equal). **Antoine Lesur**: Formal analysis (equal); investigation (supporting); resources (equal); writing – original draft (supporting). **Gunnar Dittmar**: Formal analysis (equal); writing – review & editing (supporting). **Francois Bernardin**: Investigation (supporting); writing – review & editing (supporting). **Eva Zahradnik**: Conceptualization (supporting); resources (equal); writing – review & editing (supporting). **Monika Raulf**: Conceptualization (supporting); resources (equal); writing – review & editing (supporting). **Francois Hentges**: Resources (equal); writing – review & editing (supporting). **Carsten Bindslev‐Jensen**: Resources (equal); supervision (supporting); writing – review & editing (supporting). **Markus Ollert**: Funding acquisition (equal); supervision (supporting); writing – review & editing (supporting). **Christiane Hilger**: Conceptualization (equal); funding acquisition (equal); supervision (lead); writing – review & editing (equal).

## CONFLICT OF INTEREST STATEMENT

The authors declare no conflicts of interest.

## Supporting information

Supporting Information S1

Figiure S1

Table S1

Table S2

## Data Availability

The data that support the findings of this study are available in the supplementary material of this article or are available from the corresponding author upon reasonable request.
