# Supplementary material for: Proteomic analysis of horse hair extracts provides no evidence for the existence of a hypoallergenic Curly Horse breed
Source: Clin Transl Allergy. 2024 Jan 29;14(2):e12329. doi: 10.1002/clt2.12329 (PMC10825075; doi:10.1002/clt2.12329)
Supplement: Supplementary file 1 — Supporting Information S1 [file CLT2-14-e12329-s004.docx]

**Table E1**: sIgE to horse, dog and cat dander of patient sera collected in Luxembourg

|  | Horse Dander | Dog dander | Cat dander |
| --- | --- | --- | --- |
| Serum ID | sIgE (kU/L) | sIgE (kU/L) | sIgE (kU/L) |
| 1 | >100 | 53.3 | 5.7 |
| 2 | >100 | 24.0 | 63.0 |
| 3 | >100 | 28.0 | 4.3 |
| 4 | 78 | 90.0 | 76.0 |
| 5 | >100 | 30.0 | 0.7 |
| 6 | 58 | 6.4 | 3.5 |
| 7 | 92 | 7.2 | 0.7 |
| 8 | 57 | >100 | 4.3 |
| 9 | 38 | 32.0 | 47.0 |
| 10 | 12 | 13.0 | 14.0 |

sIgE were quantified by ImmunoCAP

**Table E2**: Skin prick test results and sIgE to horse dander of patients recruited in Denmark

|  | Horse dander | |
| --- | --- | --- |
| Serum ID | SPT (mm) | sIgE (kU/L) |
| 1 | 10.5 | 69.6 |
| 2 | 11 | 69.2 |
| 3 | 6.0 | 36.5 |
| 4 | 13.5 | 25.2 |
| 5 | 10 | 18.1 |
| 6 | 10 | 13.5 |
| 7 | 4.5 | 23.4 |
| 8 | 12.5 | 13.1 |
| 9 | 13.5 | 7.1 |
| 10 | 12.5 | 4.4 |
| 11 | 3.0 | 7.7 |
| 12 | 15 | 14.8 |
| 13 | 5.0 | 5.5 |
| 14 | 4.5 | 3.3 |
| 15 | 10.5 | 9.9 |
| 16 | 10 | 6.3 |
| 17 | 7.5 | 13.5 |
| 18 | 20 | 7.6 |
| 19 | 11.5 | 0.9 |
| 20 | 10.5 | 1.4 |
| 21 | 10.5 | 3.2 |
| 22 | 5.5 | 2.3 |
| 23 | 12 | 11.0 |
| 24 | 15.5 | 2.8 |
| Mean | 10.2 | 15.4 |
| Median | 10.5 | 8.8 |

sIgE were quantified by ImmunoCAP
