# Supplementary material for: Proteomic analysis of horse hair extracts provides no evidence for the existence of a hypoallergenic Curly Horse breed
Source: Clin Transl Allergy. 2024 Jan 29;14(2):e12329. doi: 10.1002/clt2.12329 (PMC10825075; doi:10.1002/clt2.12329)
Supplement: Supplementary file 2 — Figiure S1 [file CLT2-14-e12329-s001.docx]

**A)**


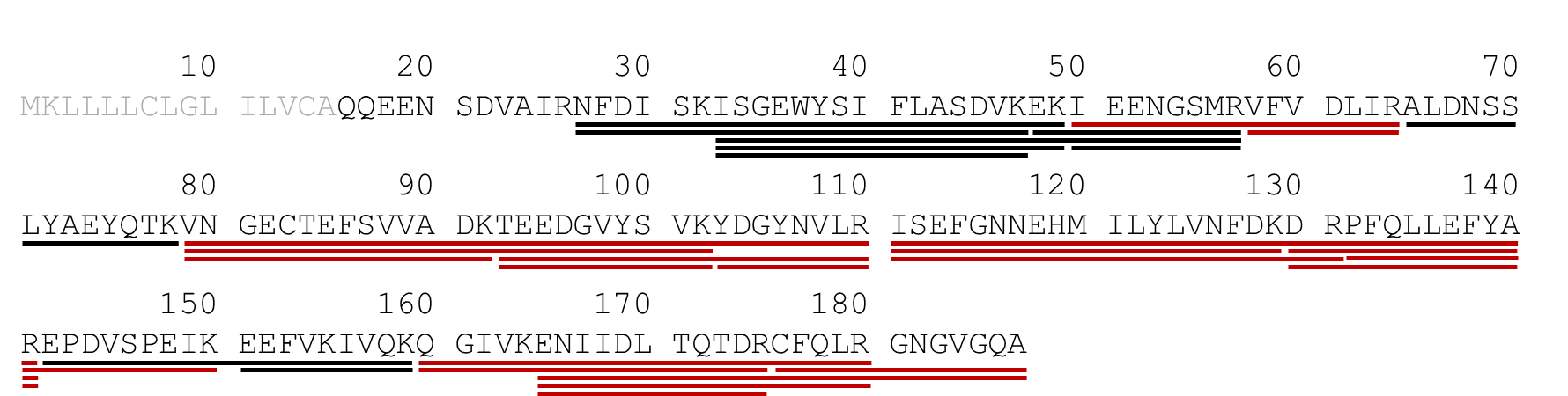


**B)**


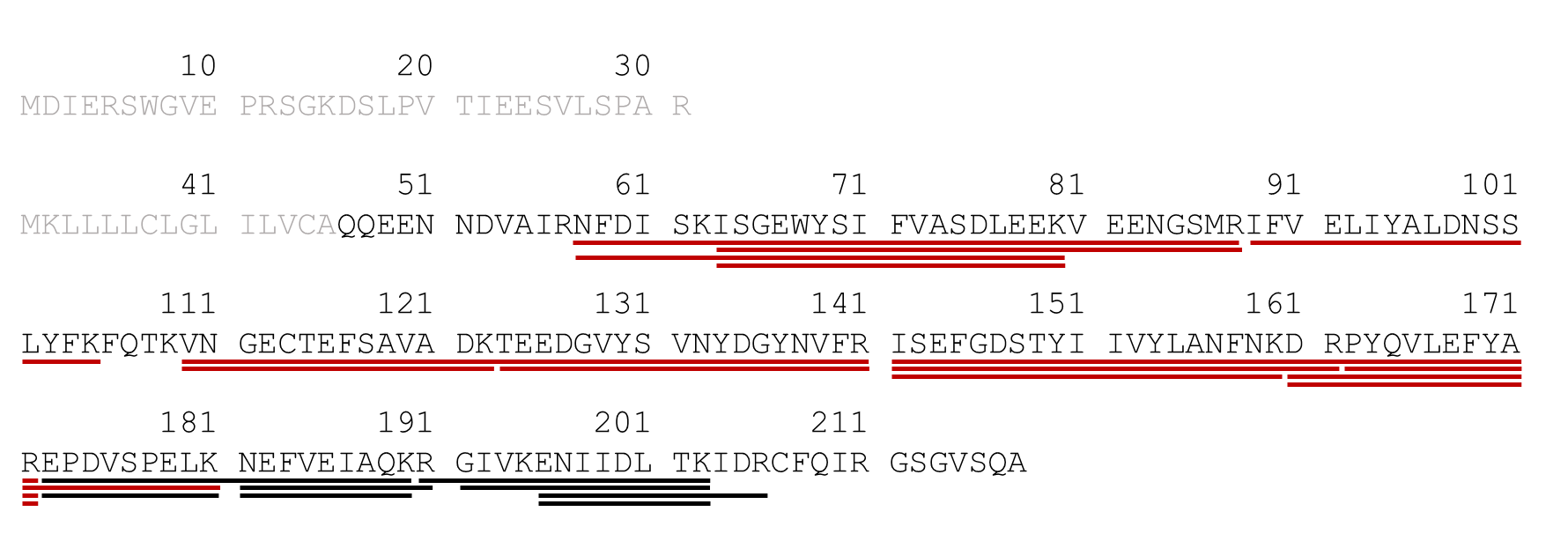


**Supplement Figure E1:** Amino acid sequence confirmation of Equ c 1 variants in horse hair extracts by LC-MS/MS. Detected peptides are shown as lines beneath the sequences. Peptides that are unique to the sequence are marked in red. Predicted pro-peptides and signal peptides are displayed in grey font. **A)** Sequence of Equ c 1.0102 (UniProt accession number: A0A3Q2HX90). In total, 30 peptides were found, of which 20 were unique to Equ c 1.0102. Peptides covered 93.6% of the mature protein sequence (172 AA). **B)** Sequence of Equ c 1.0201 (GenPept sequence reference: XP_001490299.3). In total, 22 peptides were found, of which 14 were unique to Equ c 1.0201. Peptides covered 84.3% of the mature protein sequence (172 AA).
